# Adapting to life with chronic migraine: a qualitative study of lived experiences among multi-ethnic Asian patients

**DOI:** 10.3389/fpain.2026.1825004

**Published:** 2026-07-02

**Authors:** Yasmin Idu Jion, Jun Ying Hor, Jun Zhi Teh, Yi Jing Zhao, Chai Ching Ng, Jiao Jiao Dang, Nijanth Manohararaj, Chew Seah Tan, Sungwon Yoon

**Affiliations:** 1 Department of Neurology, National Neuroscience Institute, Singapore; 2 Mount Elizabeth Medical Centre, Singapore; 3 Health Services Research and Population Health, Duke-NUS Medical School, Singapore; 4 Centre for Population Health Research and Implementation, SingHealth Regional Health System, SingHealth, Singapore

**Keywords:** Asia, burden, chronic, disability, experience, migraine, qualitative, quality-of-life

## Abstract

**Background:**

Chronic migraine is associated with greater disability, productivity losses and reduced quality of life compared to episodic migraine. Limited research has explored the multi-faceted burden of chronic migraine from patients' perspectives and experiences, particularly in the Asian context. This study aims to explore the burden of chronic migraine through the lived experience of multi-ethnic Asian patients and to identify gaps in migraine awareness, service provision and clinical management.

**Methods:**

This was a qualitative study design involving semi-structured interviews with patients with chronic migraine recruited from a Neurology outpatient clinic in a large tertiary hospital in Singapore. Data was collected through face-to-face interviews and transcribed verbatim. Inductive thematic analysis was performed to identify key themes.

**Results:**

17 participants with chronic migraine took part in the study. 4 main themes were identified which illustrate the substantial burden of chronic migraine: 1) Living with migraine long-term; 2) Dealing with stigma; 3) Navigating the healthcare journey; and 4) Regaining agency and validation. Participants described physical, psychosocial and financial consequences that disrupted their daily roles and sense of self. Stigma and the invisibility of chronic migraine compounded this burden. Participants demonstrated strong efforts to regain agency through lifestyle modifications, complementary therapies and peer support, despite limited treatment options and persistent uncertainty.

**Conclusion:**

This study provided insights into the lived experience of chronic migraine among multi-ethnic Asian patients. Our findings highlight the importance of culturally sensitive and patient-centred approaches that validate patients' experiences, address stigma, and integrate psychosocial as well as medical needs.

## 1 Introduction

Primary headaches, particularly tension type headache and migraine, are highly prevalent, affecting 3 billion individuals worldwide (1). Migraine alone impacts 14.7% of the global population, with its prevalence in Asia and Singapore estimated about 10% (2–5). According to Global Burden of Disease, migraine is the second leading cause of years lived with disability (YLD), accounting for 5.6% of all YLD globally (6). The burden of migraine has significantly escalated in recent decades, with notable disparities across regions; the greatest increase in incidence, prevalence, and disability-adjusted life years (DALYs) were observed in East Asia and Latin America (7). In Singapore, episodic migraine was estimated to cost 1 billion dollars in 2019, largely due to productivity losses (8).

Chronic migraine, defined as headache occurring on 15 or more days per month for over 3 months of which at least 8 attacks meet criteria for migraine, affects about 0.5%–5% of the global population (9). Compared to episodic migraine, chronic migraine is associated with greater disability, productivity loss, direct costs and poorer quality of life (10–12). The burden is further amplified in individuals with concurrent medication-overuse headache and comorbid anxiety and depression, both of which are not uncommon in chronic migraine (10, 13–15). Despite significant disability, chronic migraine remains poorly managed. Disease awareness and diagnosis are low across Asia, Europe and United States (US), and treatment is often inadequate (16–21). The headache community has repeatedly called for greater recognition of migraine as a serious disorder and for improvements in patient care (22).

The impact of migraine has been evaluated using quantitative tools such as Migraine Specific Quality of Life (MSQ) and Migraine Disability Assessment (MIDAS) (23). While these tools capture the effects of migraine on disability and quality of life, many personal, social, and cultural dimensions of migraine are less easily measured (24). Beyond the migraine symptoms, patients often face interictal symptoms, trigger avoidance, medication side effects, cognitive and mood changes, and comorbidities such as depression and anxiety—all of which add significantly to the burden of the disease (13, 25–30). Hence, as with any chronic pain management, migraine management should adopt a multimodal and patient-centred approach.

To date, most existing research has focused on episodic migraine in US and European populations with few studies from Asia (29, 31–33). Narrative studies can offer valuable insights into the lived experiences, capturing aspects of the condition not reflected in traditional measures (34). Yet, qualitative studies on chronic migraine are scarce, with much of the literature centred on chronic daily headaches (35–38). The long-term impact of chronic migraine also remains poorly understood.

Therefore, this study aimed to explore the lived experience of individuals living with chronic migraine and how they make sense of and adapt to the condition's consequences and burden long-term. This study sought to offer in-depth insights into patient's perspectives and approaches to managing chronic migraine, and identify potential gaps in migraine awareness, service provision and clinical management.

## 2 Methods

### 2.1 Study design

This study employed a qualitative study design involving semi-structured interviews with patients with chronic migraine.

### 2.2 Participants and setting

Eligible patients were purposively recruited according to age, gender, race, employment and years of diagnosis from the Neurology outpatient clinic in a large tertiary hospital from August 2022 to April 2023 in Singapore. English-speaking patients aged ≥21 years old and who were diagnosed with chronic migraine (defined as ≥15 headache days a month for at least 3 months, of which 8 attacks fulfil criteria for migraine) for at least one year were identified by their treating physicians during routine appointment. A study coordinator who was not involved in the care of the patient then approached the patient to invite them for the study. Non-English-speaking patients, those with clinically significant depression and anxiety or relapses as determined by the treating physicians and those unable to give written consent were excluded from the study.

### 2.2 Data collection

Prior to interviews, demographic data including age, gender, ethnicity and occupation were collected. In addition, they completed the MIDAS and Headache Impact Test-6 (HIT-6) scales, validated tools to quantify migraine disability based on number of days lost due to impaired productivity (higher scores denote greater disability) and MSQ v2.1 scale which evaluates how frequently migraine limits and prevents work, social activities, as well as the emotional impact of migraine (higher scores out of 100 denotes better health-related quality of life) (39). Depression, anxiety and Stress Scale (DASS-21) was used to assess severity of emotional states.

A semi-structured interview guide was developed with main topics including patient's overall experience and perceptions of migraine, long-term impact of migraine, coping and adaptation, and support and resources needed for better management. One-to-one interviews were conducted by qualitative research trained interviewers who were not involved in the care of the patient, either in-person in a private clinic room or virtually over Zoom. Patients were interviewed in English. Field notes were taken during the interview, and no repeat interviews were conducted. The interviews were audio recorded and transcribed verbatim without identifiers. Average interview duration was 58 min per participant. Written informed consent was taken prior to interviews.

### 2.3 Data analysis

All interviews were audio-recorded, transcribed verbatim and analysed thematically following Braun and Clarke's six-phase approach (40). The analysis was inductive and sought to capture participants' experiences of long-term migraine, challenges and adaptations. Two members of the research team (YJ, SY) independently read and coded the transcripts, generating initial codes directly from participants' accounts. Codes were compared and refined through iterative discussions among team members to build a shared coding framework. Related codes were then grouped into sub-themes and overarching themes reflecting the experiential patterns described by participants. Analysis and data collection proceeded concurrently, generating a process that influenced subsequent questions and interpretation of data. QSR NVivo was used to support data management, coding and retrieval. Reflexive memos were kept throughout the process to document analytic decisions and enhance transparency. To ensure rigor and transparency, this study was guided by the Consolidated Criteria for Reporting Qualitative Research (COREQ) (41).

### 2.5 Ethics approval

All procedures were performed in compliance with relevant laws and institutional guidelines and have been approved by the appropriate institutional committee (SingHealth Centralized Institutional Review Board CIRB Ref: 2022/2299). Written informed consent was obtained from all participants.

## 3 Results

In total, 30 patients were approached, of whom 17 consented and were interviewed. Data saturation was reached at 17 participants. Those who declined cited time constraints or discomfort discussing their experiences. Participants had a mean age of 43.9 years (range: 21–61), mean headache frequency 21.9 days (range 16–30 days), 65% were female and 76% were employed. Mean MIDAS score was 56, HIT-6 score was 64, and MSQ scores were as follows: Role Function-Restrictive (RR) 43, Role Function-Preventive (RP) 58 and Emotional Function (EF) 44. **(**Table 1**)**.

**Table 1:** Participant characteristics.

Participant No.	Age	Race	Gender	Employment	HIT6	MIDAS	DASS stress	DASS anxiety	DASS depression	MSQ RR	MSQ RP	MSQ EF
1	52	Chinese	Female	Unemployed	Substantial impact	I	Normal	Normal	Normal	37	55	53
2	36	Chinese	Female	Part time	Severe impact	IV	Normal	Moderate	Normal	34	45	66
3	45	Chinese	Male	Full Time	Severe impact	IV	Normal	Normal	Mild	28	65	13
4	37	Chinese	Female	Full Time	Severe impact	III	Normal	Normal	Normal	57	70	47
5	21	Chinese	Female	Unemployed	Severe impact	III	Normal	Normal	Normal	51	75	20
6	34	Chinese	Female	Full Time	Severe impact	IV	Normal	Mild	Moderate	54	80	67
7	55	Chinese	Male	Full Time	Substantial impact	I	Normal	Normal	Normal	80	90	100
8	57	Chinese	Male	Full Time	Severe impact	IV	Normal	Mild	Normal	17	30	53
9	43	Chinese	Female	Unemployed	Severe impact	I	Normal	Moderate	Mild	46	50	60
10	45	Chinese	Female	Full Time	Severe impact	IV	Normal	Normal	Normal	71	95	80
11	47	Malay	Female	Unemployed	Severe impact	IV	Normal	Normal	Mild	14	25	13
12	55	Caucasian	Female	Part time	Severe impact	III	Normal	Normal	Normal	40	70	47
13	40	Malay	Female	Full Time	Severe impact	IV	Normal	Moderate	Mild	49	50	20
14	47	Chinese	Male	Full Time	Severe impact	IV	Mild	Moderate	Mild	31	55	13
15	45	Malay	Male	Full Time	Substantial impact	III	Normal	Mild	Mild	42	50	20
16	61	Malay	Female	Part time	Severe impact	IV	Normal	Normal	Normal	20	15	20
17	27	Chinese	Male	Full Time	Severe impact	IV	Mild	Mild	Mild	54	65	60

We identified 4 main themes: living with migraine long-term; dealing with stigma; navigating the healthcare journey; and shifting from survival to stability **(**Table 2**)**.

**Table 2:** Themes and subthemes.

Themes	Subthemes
Living with migraine long-term	More than a headache
	Balancing symptoms and consequences of treatment
	Missing out on responsibilities
	Hopelessness: impaired psychological wellbeing
Dealing with stigma	Invisible illness
	Feeling judged
	Pressure to continue to perform
Navigating healthcare journey	Misaligned expectations and goals of care
	Cultural influences and non-medicinal approaches
	Long-term financial burden of illness
	Empathy through communication
Regaining agency and validation	Hoping for a sense of control
	Not alone: seeking peer support and validation
	Desire to be believed through public education

### 3.1 Theme 1: living with migraine long-term

Participants' narratives illustrated that migraine was a chronic experience with recurrent flares that went far beyond a headache. Participants described having to deal with non-headache symptoms such as sensory hypersensitivity, vertigo and numbness, and these were as debilitating as the pain.

I'll be vomiting, and I can't have any lights or any sound at all. I need to basically lock myself into the room with darkness, curtain, window shut (#P3).

My balancing is affected…somehow, I feel like I have lost gravity. I may be walking… suddenly I feel somebody pushing me… like when strong wind coming, I feel like I'm being gushed away (#P10).

Migraine management was a continual weighing of potential benefits against unwelcome consequences of the medication. Patients constantly negotiated between the disabling impact of their symptoms and drawbacks of the medication. For some, medication was essential to maintain function, yet sedating side effects imposed a different kind of impairment. Social plans were often cancelled to avoid the adverse effect of the medication.

If I don't take (sumatriptan), it will definitely affect my function. But the medication makes me drowsy. I usually have to lie down (with the medication) and not do anything and try to get some sleep (#P16).

If it's a social call, like, 80% of the time I will cancel it, so that I don't have to take any medication. If it's like work or school, then I will take just in case (#P6).

Long-term chronic migraine emerged not only as a personal health burden but also as a source of lost roles and responsibilities. Participants described how migraine disrupted their core identities as employees, parents or family members, evoking feelings of guilt, sacrifice and diminished self-worth.

I told my boss about my condition. Fortunately, my boss is understanding, she didn't give me major projects to run. She doesn't want to put me in a big scale project (#P14).

I am usually the one to drive them everywhere.playdates, classes, etc. But then I got this sickness, I can't drive them..So, I feel like I did not actually do a good mom's role for them (#P10).

Participants' accounts also revealed deep psychological toll of living with recurrent, unpredictable migraine attacks. The invisible and seemingly incurable nature of the condition intensified feelings of “hopelessness' during attacks. The perception of “no cure” generated fears about the future and a sense that there is “no light at the end of the tunnel.”

I was thinking of how am I going to cope with the future when it's already so bad? Definitely not easy for lot of moments. I also can say that I break down quite a lot. I sometimes don't really know what I can do also (#P17).

I went for a CT scan and MRI, there's nothing wrong with my brain. But I still suffer whenever there's an attack. It's almost like a terminal illness, that there's no cure, you don't see anything at the end of the tunnel. There's no light at the end of the tunnel (#P13).

### 3.2 Theme 2: dealing with stigma

Participants commonly mentioned the largely hidden symptoms of migraine led to misunderstanding by close and distant others who often dismissed the condition as exaggerated or imagined.

They (Family) all feel like it is my imagination. Because I look normal, people will see like you are so normal, but they don't know inside you are struggling (#P10).

People do not understand how bad it is. They really don't see you as someone who is handicapped or anything. You just look so normal (#P12)

Participants described how they were morally judged by people in their social and work environments and even by healthcare providers who accused them of malingering, drug-seeking or attention-seeking.

The doctors, unfortunately, were not very kind. They say that I can't be having that much pain, and I was trying to skip school (#P2).

I think being dismissed is the biggest challenge. They (doctors) thought I was addicted. But I only take it when I'm in pain. And you don't know me, you can't accuse me of being addicted when you don't understand my pain. Getting them to take me seriously took a lot of effort. I don't know why, but they treat me like I am lying (#P6)

They don't understand this guy, is he slacking or is he just giving excuses? Most of them do not understand that this thing actually paralyzes me. So, when I said I just couldn't do it they thought I'm just procrastinating my work (#P15).

Despite significant pain and suffering, participants felt compelled to suppress symptoms and maintain productivity to meet workplace demands and expectations. They described routinely taking painkillers and pushing on with tasks or isolating themselves while continuing to work through attacks.

Work will still go on because work is my first priority. I will go down or I will look for any GP for painkiller injection then I will go back to work again (#P14).

I will just go to one place and avoid people for a bit so that I can calm myself down, because when I do get migraine, I get a bit agitated… I will quickly reach out for the painkiller. And if I can tahan (cope), I will still be at work. (#P15).

### 3.3 Theme 3: navigating healthcare journey

Participants depicted how the chronic, relapsing nature of migraine and the limits of treatment created a persistent gap between what they hoped for and what they actually experienced. This mismatch resulted in disappointment and resignation. While some came to accept that medications did not “work magic”, others remained frustrated.

I am not happy with it [medicine], but you have to come to terms with it. I mean… it doesn't work perfectly like 100% taking away the pain or the medicine does not have an immediate effect. The medicine doesn't work magic (#P8).

So far no one can help me. If they (doctors) cannot cure me, they should help me with the frequency of it…I am not happy with 50% reduction of [symptom] frequency. I think 50% is still not good enough for me. It's better than nothing but I'm not happy with 50% (#P16).

When conventional treatments were perceived as insufficient, participants turned to multiple non-medicinal approaches such as traditional Chinese medicine or alternative therapies, often influenced by family members or cultural beliefs. Experiences with these approaches varied: some provided calming relief while others worsened symptoms.

I actually went TCM before, but I don't see a relief. Then, I went to a chiropractor for the cervical adjustment, but I feel even more dizzy after that. I also tried yoga therapy—Ayurveda. I think it helps to calm your body (#P10).

I saw a healer. When western medicine cannot help me then it's no longer a normal headache, it could be something else because they (family) believed in spirits and all these. There is a disturbance there that's why western medicine cannot help (#P16).

Unquestionably, managing chronic migraine imposed a substantial and ongoing financial burden. Participants reported that even when effective treatments were available, affordability remained a critical barrier. Subsidies or financial assistance schemes provided some relief, but out of pocket payments were still frequently required.

Cost is a big issue. You cannot even afford some medications. Even if it works, it doesn't make sense. It is too expensive. Majority will not be able to afford (#P8).

I'm thankful to know that I can apply for Medifund and then the government subsidize the medication, but there are certain medications that I have to come out with the payment (#P11).

Despite multiple challenges, participants spoke of the value of doctors who listen, validate experiences and explain treatment processes in their long-term healthcare journey. The presence of supportive clinicians enabled them to feel comfortable discussing their condition and making informed treatment choices.

My doctor didn't dismiss me at all…She explained to me the process, why we cannot immediately go full dose…She listens. And she's willing to discuss with me (#P6).

### 3.4 Theme 4: regaining agency and validation

Through the interviews participants strived to regain a sense of agency over a condition that they found deeply debilitating. They consistently spoke about resisting the urge to give in and pushing through. The importance of self-control, discipline and life regulation was highlighted as a way to mitigate and cope with migraine triggers. However, even with substantial efforts, achieving full control seemed elusive.

It is the management of your own lifestyle. You really need a lot of discipline. If you want to manage migraine, you should be managing your lifestyle and your own habits and to manage your external stimuli (#P8).

…let's say migraine is triggered by stress. We really have to do something to try changing the source, try to control…I've been trying to work on it for so many years, but it is still work in progress. It's not easy (#P15).

Seeking out online communities and forums and engaging with others who understand the severity of migraine helped alleviate feelings of isolation. These spaces also allowed participants to share strategies for treatment, coping and alternative treatment.

Mostly is just to seek for validation because I guess it is nice to have someone just say things will get better soon. It is also nice to see that people got relief after many years of migraine, so there is a form of hope (#P5).

Even though I've been having migraine for so long, I get some sort of assurance that there are so many others like me, and they are also seeking treatment…. I just want to know how are they managing? I feel a bit comforted that others are also in pain. (#p15).

Participants expressed a strong desire for society to recognize migraine as a serious and multifaceted condition. They mentioned how in their lived experience, the absence of public understanding compounded feelings of isolation. Media campaigns, television features and public messaging were suggested not merely as informational tool but as a means of fostering empathy and alleviating the social marginalization they experienced.

More public awareness that migraine can be very broad, there are many different types of migraine. It will be good to bring in some awareness that migraine is not just a normal kind of headache (#P10)

## 4 Discussion

This study provides the in-depth explorations of the lived experience of chronic migraine among multi-ethnic Asian patients. By examining how participants navigate life with a long-term invisible condition, our findings extend existing evidence from Western contexts and highlight culturally specific challenges and strategies in managing migraine.

### 4.1 Key findings

Our findings illustrate the substantial burden of chronic migraine on the daily lives of Asian patients. Chronic migraine extended beyond headache pain to encompass non-headache symptoms, medication dilemmas and disruptions to physical and psychological wellbeing. Similar to the literature on chronic daily headache, our participants reported aura, nausea, sensory sensitivities and medication-related fears that contributed to disability and lifestyle restriction (35, 38). They frequently sacrificed social activities to minimize triggers and avoid medication, echoing prior findings on lost roles and responsibilities in work, caregiving and social life (42, 43). The emotional toll was profound, with recurrent feelings of helplessness, guilt and hopelessness, in line with evidence from other qualitative and narrative reviews on migraine and its psychological impact (27, 31–33, 44).

Participants' accounts underscored the stigma inherent in living with an invisible and unpredictable illness. Consistent with Rutberg's description of “being obliged to endure life accompanied by an unpredictable and invisible disorder,” patients in our study felt doubted by others, judged as exaggerating or seeking secondary gain, and perceived as less deserving of workplace (45). The added shame of suffering from an invisible condition amplifies the burden of migraine (35). The invisibility of migraine and the minimisation of its severity by employers, families and even health professionals amplified patients' shame and isolation, highlighting an urgent need for de-stigmatisation at public, institutional and clinical levels (46, 47).

Our findings also highlight the complex healthcare journey for patients with chronic migraine. Participants reported trying a wide range of complementary and alternative therapies—such as traditional Chinese medicine, Ayurveda, spiritual healers and chiropractic care—reflecting cultural influences and family advice, as seen in other multi-ethnic contexts (48). Despite subsidies, many faced significant out-of-pocket costs for consultations and medications, creating a persistent financial burden and limiting access to effective therapies. Participants frequently encountered a mismatch between their expectations and treatment outcomes, leading to frustration and disappointment when cure was not achieved. Endpoints traditionally used in migraine therapeutic trials (e.g., change in baseline in monthly headache/migraine days, ≥50% responder rates) were not deemed to be sufficient for patient satisfaction. Suggested alternative endpoints that emphasize outcomes that are patient-centric include Patient Global Impression of Change, treatment satisfaction measures, in addition to quality of life and physical functioning scales should be considered. As in Cotrell's and Peters' work, participants emphasized the importance of collaborative, respectful communication with physicians, feeling listened to, and having management plans tailored to their needs (38, 49). Supportive doctor–patient relationships were perceived as key to validating their experiences, dispelling misinformation, and improving adherence to prophylaxis as shown in other studies (50–52).

Finally, participants described striving to regain agency over a condition that felt unpredictable and disabling. They actively modified lifestyles, regulated stress and sought information to understand their triggers, reflecting prior study on balancing acceptance with control (53). While trigger avoidance may prevent or lessen migraine attacks, there is debate that they may contribute to further disability if avoidance is done excessively, perpetuating fear-avoidance behaviours and widening social restrictions. Many turned to social media and online communities to access information, share experiences, and gain reassurance that their struggles were real, similar to findings by a study on peer support for invisible illnesses (31, 54). Such networks not only validated their symptoms but also offered stories of hope and recovery. Participants expressed a strong desire for broader public education on migraine through media campaigns and awareness initiatives to improve societal understanding, reduce judgement, and strengthen advocacy for themselves and others living with chronic migraine.

### 4.2 Implications

Our findings have several implications for practice. Clinicians should proactively assess the psychosocial as well as physical impact of long-term chronic migraine, validate patients' experiences, and adopt shared decision-making to align treatment goals with patient priorities. Incorporating culturally sensitive education on medication use, lifestyle modification and complementary therapies may help patients feel supported in their self-management journey. At the system level, policies to improve affordability, public education and workplace accommodations are needed to reduce stigma and financial strain. Together, these steps could foster greater empathy, strengthen doctor–patient partnerships and support patients in regaining agency over their lives with chronic migraine.

### 4.3 Strengths and limitations of the study

This study represents the first qualitative study of chronic migraine conducted in an Asian context. Its strengths include a diverse sample drawn from a multi-ethnic population and concurrent collection of disability and quality of life measures (e.g., MIDAS, HIT6 and MSQ2.1), which highlighted how quantitative scores may underestimate the burden of disease conveyed in patients' narratives. We also limited recruitment to patients with at least one year of chronic migraine to reduce fluctuations between high frequency episodic and chronic migraine. Limitations include the exclusion of non-English speaking patients, owing to interviewers' lack of dialect proficiency, and this may inadvertently under-represent certain subgroups of patients. Nevertheless, most of our patients were literate in English. We also excluded clinically significant depression and anxiety to minimise the added burden contributed by relapses of these mood disorders, but have included stable depression and anxiety. We acknowledge that depression and anxiety are strong comorbidities with chronic migraine and may limit generalizability to the broader population. Of the 30 patients approached, 17 consented to participate. This raises the possibility of selection bias. While this may limit the findings' transferability, the sample size is comparable to other qualitative studies, and data saturation was achieved (55). While patients were recruited from a single centre, this centre is the national centre for neuroscience for Singapore and accepts referrals from throughout the country. Our study also did not take into migraine subtypes (with and without aura), the presence of concomitant medication overuse headache and treatment data, and we acknowledge that this may shape the narrative experience of our patients. In particular, insights to the access or use of newer preventive therapies could provide novel findings in the current therapeutic landscape of chronic migraine and could be the direction for future research.

## 5 Conclusion

This study provided insights into the lived experience of chronic migraine among multi-ethnic Asian patients. Beyond pain, participants described far-reaching physical, psychological, social and financial consequences that disrupted their daily roles and sense of self. Stigma and the invisibility of migraine further compounded this burden, underscoring the need for greater public awareness and more empathic healthcare encounters. Participants' accounts also revealed strong efforts to regain agency through lifestyle modification, complementary therapies and peer support, despite limited treatment options and persistent uncertainty. These findings highlight the importance of culturally sensitive, patient-centred approaches that validate experiences, address stigma, and integrate psychosocial as well as medical needs. Improving affordability, access to care and public education may ultimately help patients achieve better quality of life and greater control over their condition.

## Data Availability

The raw data supporting the conclusions of this article will be made available by the authors, without undue reservation.

## Supplementary material

The Supplementary Material for this article can be found online at: https://www.frontiersin.org/articles/10.3389/fpain.2026.1825004/full#supplementary-material

Supplementary Consolidated criteria for reporting qualitative research (COREQ) checklist
